# Longitudinal survey of total airborne bacterial and archaeal concentrations and bacterial diversity in enriched colony housing and aviaries for laying hens

**DOI:** 10.1016/j.psj.2024.104119

**Published:** 2024-07-30

**Authors:** Magali-Wen St-Germain, Valérie Létourneau, Perrine Cruaud, Candice Lemaille, Kim Robitaille, Éloïse Denis, Martine Boulianne, Caroline Duchaine

**Affiliations:** ⁎ Department of Biochemistry, Microbiology and Bioinformatics, Université Laval, Québec, Canada; † Research centre of Quebec Heart and Lung Institute, Quebec, Canada; ‡ Independent Researcher, Lourenties, France; § Faculty of Veterinary Medicine, Université de Montréal, Saint-Hyacinthe, Canada

**Keywords:** bioaerosol, bacteria, archaea, aviary, enriched-cage

## Abstract

Conventional cages for laying hens will be banned in Canada as of 2036, and the egg industry is transitioning toward enriched colony housing and aviaries. While higher concentrations of particulate matter have been previously reported in aviaries and other cage-free housing systems, concentrations of total bacteria and archaea suspended in the air are still uncharacterized in Canadian enriched colonies and aviaries. The aim of the present study was to conduct a longitudinal survey of airborne total bacteria and of airborne total archaea in twelve enriched colonies and twelve aviaries in Eastern Canada during a whole laying period. High-throughput sequencing of 16S rRNA gene amplicons was used to reveal and compare bacterial diversity at the start and the end of the production cycle, and during the cold and the warm seasons. Total bacterial and archaeal concentrations were significantly higher in aviaries (*p* < 0.05) versus enriched colonies, and in the cold season for both housing types (*p* < 0.05). While flock age did not have a significant effect on total bacterial and archaeal concentrations, it did on bacterial diversity in both enriched colony houses and aviaries (*p* < 0.05). The 2 housing systems were significantly different in their diversity of bacteria.

## INTRODUCTION

Raising awareness about laying hen welfare led to new requirements for housing systems in Europe and in some American states (Appleby, 2003; Shields et al., 2017). In Canada, the egg industry has mandated a gradual phase out of conventional battery cages and a transition toward alternative housing systems for laying hens, such as enriched colonies and cage-free housing systems (National Farm Animal Care Council and Egg Farmers of Canada, 2017).

While alternative housing systems provide more freedom of movement and enrichments for the animals (i.e. perches, recluse nesting areas, access to litter) (Appleby and Hughes, 1991; Duncan et al., 1992; Tanaka and Hurnik, 1992; Hartcher and Jones, 2017), studies suggest cage-free houses may face more challenges regarding infectious diseases and parasitic control than houses with cages. Cage-free housing systems with access to a litter floor have also been associated with higher airborne dust concentrations than cage-houses (Nimmermark et al., 2009; Le Bouquin et al., 2013; Arteaga et al., 2015). In poultry operation, dust particulates mainly originate from the animals (e.g., skin, feces), from the litter and from the feed (Ellen et al., 2000; Kirychuk et al., 2006; Just et al., 2009; Cambra-López et al., 2010). Dust particles suspended in the air are known to cause irritation of the respiratory tract and decreased immune response in poultry (Wang et al., 2023a). While occupational exposure to airborne bacteria often leads to non-infectious symptoms in workers, such as airway inflammation and allergic reactions (Donham et al., 2000; Viegas et al., 2013), and some bacteria found in poultry facilities are identified pathogenic agents for both fowls and humans (ex.: *Salmonella* sp., *Escherichia coli, Campylobacter jejuni, Enterococcus* sp.) (Chinivasagam et al., 2009; Just et al., 2011; Just et al., 2012; Lawniczek-Walczyk et al., 2013).

Concentrations of airborne bacteria found in poultry operations are known to be influenced by manure management practices, feed type and handling, ventilation rates, seasons, presence of bedding material, animal activity and flock age (Calvet et al., 2009; Nimmermark et al., 2009; Ellen et al., 2000). Previous studies have reported total airborne bacterial concentrations of 10^7^–10^8^ cells or copies/m³ in both broilers and laying hen facilities (Radon et al., 2002; Oppliger et al., 2008; Nimmermark et al., 2009; Just et al., 2011) and the dominant phyla being *Firmicutes, Bacteroidetes, Proteobacteria, Cyanobacteria, Fusobacteria* and *Actinobacteria* (Chen et al., 2022; Cui et al., 2023).

Dominant genera in bioaerosols of laying hen houses were reported to be Lactobacillus, unknown Bacteroidetes, Turicibacter, Facklamia, Corynebacterium, Fusobacterium, Aerococcus, Comamonas, Faecalibacterium, Enterococcus, Olsenella, Joetgalicoccus and Stenotrophomonas (Cui et al., 2023).

While bacterial content has been evaluated, fewer studies have explored airborne archaeal concentrations and diversity in poultry facilities. Archaea have been detected by molecular methods in various livestock facilities, including dairy cows barns (Blais-Lecours et al., 2012), in swine barns (Nehmé et al., 2009; Létourneau et al., 2020), broilers and laying hen operations (Just et al., 2013), with concentrations from 1.2 × 10^4^ to 1.2 × 10^6^ copies/m³. While the health effects from the exposure to airborne archaea remain poorly characterized, the intranasal exposure of the 2 methanogen species *Methanobrevibacter smithii* and *Methanosphaera stadtmanae* led to inflammatory response in a murine model (Blais-Lecours et al., 2011). Archaea suspended in the air might thus contribute to the development of respiratory symptoms among producers exposed to bioaerosols of laying hen facilities.

Multi-tiered cage-free housing systems for laying hens (aviaries) offer more freedom of movement for animals than enriched colony housing, and access to a litter floor for the hens to forage, dust bathe and scratch. Consequently, aviaries may provide suitable conditions for more aerosolization of bacteria and archaea. Lower ventilation rates may result in higher bacterial and archaeal concentrations as well. Changes in the gut microbiota of laying hens (Dai et al., 2022; Cui et al., 2023) may translate into variation in bacterial diversity in bioaerosols, as feces are a source of bioaerosols (Cambra-López et al., 2010).

Since no study has ever compared the bacterial and archaeal concentrations in aviaries and enriched colonies, and since the 2 types of housing systems will become essential in Canada. The aim of this project was to undertake a longitudinal survey on the levels of both total airborne contaminants in these housing systems, through several weeks during the production cycle, the effects of the housing type, the flock age and the seasonal effect. Additionally, the bacterial diversity was assessed at the start and at the end of the production cycle, and during both seasons. Results of the present project may give insight on the effect of 2 alternative housing systems for laying hens on airborne bacteria and archaea and the possible factors influencing their concentrations and diversity.

## MATERIAL AND METHODS

### Sampling of Bioaerosols

Twelve aviaries and twelve enriched colonies (using enriched cages) housing between 10,000 and 50,000 hens and located within 260 km from the Faculty of Veterinary Medicine at Université de Montréal (Saint-Hyacinthe, Québec, Canada) were visited for this project (October 2017 to October 2021). All facilities housed Lohmann LSL-Lite white hens, with the exception of one aviary and one enriched housing system had Dekalb white hens. Every barn had monthly visits during the first five months of the production cycle (19, 23, 27, 31, and 35 wk of life, or closest to these ages) and at 45, 55, and 65 wk of life. For each visit, 3 air samples were simultaneously collected on polycarbonate filters (0.8 µm porosity; SKC Inc., Eighty four, PA) in 37 mm cassettes at a flow of 2 L/min (Gilian GilAir-5 Air Sampling Pumps; Sensidyne, St. Petersburg, FL) for 120 min (0.24 m³ of air per sample). Pumps were calibrated using the TSI 4043 Mass Flow Meter (TSI, Shoreview, MN). A field blank was done at every visit. Filters were kept at 4°C until processing. Sample processing and DNA extraction were performed during June to August 2020, June to August 2021, and November 2021.

### Sample Treatment and DNA Extraction

Cassettes were conditioned at room temperature before recovering bioaerosols by adding 4 mL of phosphate-buffered saline 1X (PBS, 1X) sterile solution, supplemented with 0.05% Tween20 into each cassette. They were then shaken using the orbital shaker VWR Digital 3D Rotator Waver (VWR, Radnor, PA) for 30 min at ambient temperature. The eluted bioaerosols per cassette were recovered and aliquots of 1 mL were centrifuged at 21,000 × *g* for 10 min, and the supernatant discarded. Pellets were kept at -20°C until DNA extraction using the DNeasy PowerLyzer PowerSoil Kit by QIAGEN (Germantown, MD) according to the manufacturer's instructions. Extracted DNA was stored at -20°C until further analysis.

### qPCR Analyses

Quantification of total bacteria and archaea was done using the primers and probes shown in Table 1. For total bacteria, 2 µL of extracted DNA were added to the reaction mix: 10 µL of iQ Supermix, 0.1 µL of forward and reverse primers (250 nM of each), 0.1 µL of probe (50 nM), and 7.8 µL of sterile nuclease-free water, totaling a volume of 20 µL. The PCR protocol consisted of 94°C for 3 min, followed by 40 cycles of 95°C for 20 s and 62°C for 1 min. For total archaea, 2 µL of extracted DNA were added to the mix containing 10 µL of iQ™ SYBR® Green Supermix, 0.1 µL of forward and reverse primers (250 nM of each), and 7.9 µL of sterile nuclease-free water for a total volume of 20 µL. The PCR protocol was conducted accordingly: 95°C for 3 min, next were 35 cycles of 95°C for 10 s, 55°C for 20 s, and 72°C for 25 s, then a melting curve analysis was done to confirm the specificity of the SYBR Green fluorescence.

**Table 1:** Primers and probes used for the qPCR assays.

qPCR assays	Targets	Sequences (5′–3′)	References
Total bacteria	V4–V5 region of 16s rRNA genes	F: GGTAGTCYAYGCMSTAAACG R: GACARCCATGCASCACCTG P: /56- FAM/TKCGCGTTG/ZEN/CDTCGAATTAAWCCAC/ 3IABkFQ/	(Bach et al., 2002)
Total archaea	V4 region of 16s rRNA genes	F: CCGACGGTGAGRGRYGAA R: YCCGGCGTTGAMTCCAATT	(Baker et al., 2003)

Both thermal cycling protocols were done using the CFX384 Touch Real-Time PCR Detection System (Bio-Rad, Hercules, CA), with non-template controls being performed during each individual assay. Standard curves were produced with plasmids containing the targeted sequence of the qPCR assays. Plasmids, primers and probes were purchased from Integrated DNA Technologies (IDT, Coralville, IA). Data were expressed in gene copies/m³ of air.

### Statistical Analysis for qPCR Data

Data were analyzed using R (version 4.0.3). As they did not follow a normal distribution, a generalized linear mixed model (**GLMM**) from the lme4 package was used. The poultry facility was defined as a random factor, while the following elements were set as explanatory variables: the housing type (enriched cages or aviaries), season (cold or warm), months, and flock age (in weeks). Based on local climate normals^1^, the cold season involved samples collected from October to April, inclusively, for average outdoor temperatures below 10°C. As for the warm season, it included the months of May to September, for average outdoor temperature above 10°C.

The statistical analyses using generalized linear mixed models (**GLMM**) with a gamma distribution (R, v.4.0.3, package lme4) were undertaken to assess the contribution of each explanatory variable on total bacterial and on total archaeal concentrations, and the possible interactions between all these (α: 0.05).

### Analysis of Bacterial Diversity by High-Throughput Sequencing

Specific samples from each facility were selected for high-throughput sequencing. Triplicates were pooled in equimolar ratios for diversity analysis of samples taken: at the start (visit at 19 or 23 wk of aging flocks, or the closest), and at the end of the production cycle (visit at 55 or 65 wk of aging flocks, or the closest), during the warm (visit at 35 or 45 wk of aging flocks, or the closest), and cold season (visit at 35 or 45 wk of aging flocks, or the closest). For each facility, field blanks from every single one of these sampling periods were pooled before being sequenced.

High-throughput sequencing was performed at the *Plateforme d'analyse génomique (Institut de biologie intégrative et des systèmes*, Université Laval, Québec, QC, Canada), including amplification of target sequences and sample pooling in equimolar ratios. Illumina amplicon sequencing comprises of the flowing steps: library preparation, cluster amplification, sequencing and data analysis (Mardis, 2008; Liu et al., 2012). Cluster amplification and sequencing are performed on a height-channel microfluidic cell.

### Library Preparation

Library preparation was conducted using a 2-step protocol with dual indexing designed for Illumina instruments (CA). An initial PCR reaction was carried out with a specific primer set targeting the V6 and V8 variable regions of the 16s rRNA gene (Comeau et al., 2011) customized for Illumina TruSeq (Table 2), using the following reaction mix: 1× Q5 Buffer (NEB, Ipswich, MA), forward and reverse primers at a concentration of 0.25 µM each, dNTPs at a concentration of 200 µM each, 1 U of Q5 High-Fidelity DNA Polymerase (NEB, Ipswich, MA) and 1 µL of DNA extract, for a total volume of 25 µL per run. The PCR protocol consisted in heating at 98°C for 30 s, followed by 35 cycles of 98°C for 10 s, 55°C for 10 s, 72°C for 30 s and a final extension at 72°C for 2 min. PCR products were then purified with the Axygen AxyPrep MAG PCR Clean-Up Kit (MA) and quality was verified on a 1% agarose gel. The second PCR for library preparation was performed with 50 to 100-fold dilutions of the earliest products. It was conducted to add nucleotides complementary to the flow cell's, which will allow the binding of the amplicons onto the flow cell for the upcoming steps, and to add the molecular indexes at the extremities of the amplicons (Table 2) (Mardis, 2008; Liu et al., 2012; Cruaud et al., 2017). Indexes may be used for *in silico* sorting of sequences in multiplex assays (Cruaud et al., 2017). A 12-cycle PCR included the same steps as the previous one was then carried out, followed by purification with the PCR clean-up kit from Axygen (MA). Quality controls were run on DNA 7500 chips for Bioanalyzer (Agilent, Santa Clara, CA) and quantified by spectrometry using the NanoDrop ND-1000 (Thermo Fisher Scientific, Waltham, MA).

**Table 2:** Primers used for amplifying the V6 and V8 regions of 16s rRNA genes for analysis of bacterial diversity by high-throughput sequencing.

Primers1	Sequences (5′–3′)
1st set of PCR primers	F: ACACTCTTTCCCTACACGACGCTCTTCCGATCTACGCGHNRACCTTACC R: GTGACTGGAGTTCAGACGTGTGCTCTTCCGATCTACGGGCRGTGWGTRCA
2nd set of PCR primers	F: AATGATACGGCGACCACCGATCTACA[index1]ACACTCTTTCCCTACACGAC R: CAAGCAGAAGACGGCATACGAGAT[index2]GTGACTGGAGTTCAGACGTGT

1Primers used in the present study are Illumina-specific sequences protected by intellectual property (Oligonucleotide sequences © 2007–2013 Illumina, Inc. All rights reserved. Derivative works created by Illumina customers are authorized for use with Illumina instruments and products only. All other uses are strictly prohibited.)

Subsequent clustering amplification and sequencing steps were performed as recommended by the manufacturer (Mardis, 2008; Liu et al., 2012).

### Bioinformatics Pipeline and Statistical Analysis

Demultiplexed data were analyzed for quality using FastQC (v.0.11.9) and Vsearch (v.2.21.1). Forward and reverse reads were then merged with Vsearch (v.2.21.1), with default settings and a minimum overlap of 10 base pairs (bp). Primer sequences (17 nucleotides at the start and 21 nucleotides at the end) were trimmed contigs using Cutadapt (v.3.5). Sequences were then filtered to keep a minimal 390 bp and a maximum 470 bp in length. These were then dereplicated and clustered into Operational Taxonomic Unit (**OTU**) with 97 % similarity. Singleton and possible chimeric OTUs were removed from the data set (*de novo* chimera detection).

Taxonomic assignments of OTUs were performed using the Mothur (v.1.47.0) (Schloss et al., 2009) version of the “Bayesian” classifier on the Silva database (release 138.1, https://www.arb-silva.de). The OTU table was acquired for the 2nd to the 8th taxonomic ranks. OTUs affiliated with “unclassified”, “Archaea”, “mitochondria”, “chloroplasts” and “Eukaryota” were eliminated from the data set.

OTUs identified in any blank (PCR or field blanks) were removed from their maximum number in the corresponding samples, being rarefied to 2,041 sequences. OTU assignment was made using the previous designation for the phylum *Bacillota* (*Firmicutes)* (Oren and Garrity, 2021) and will be referred as such herein. However, the newest term *Bacteroidota* is used to describe the previous designation *Bacteroidetes.*

All statistical analyses (Shannon diversity index, Bray-Curtis dissimilarity, non-metric multidimensional scaling [NMDS], non-parametric multivariate analysis of variance [NPMANOVA], Student's t-test) were conducted in the R software environment (v.4.2.1) using the RStudio toolkit (v.2023.06.1.524) implemented with the *Vegan* package (Oksanen et al., 2007). Species richness and the Shannon index were useful for alpha diversity analysis. Comparisons among housing types were performed with a non-parametric equivalent to the Student's t-test (Mann-Whitney U test), while that among aging flocks (start/end of the production cycle) and seasons (cold/warm) for each housing setup were with the Wilcoxon test. Bray-Curtis dissimilarity index was calculated for all pairs of samples. NMDS was then used to produce an ordination plot based on these indices and NPMANOVA to determine significant differences between corresponding clusters of samples. SIMPER (similarity percentage) analyses were for determining the contribution of each microbial group to the Bray-Curtis index (Clarke, 1993).

## RESULTS

### Total Airborne Bacterial Concentrations

**Figure 1:**
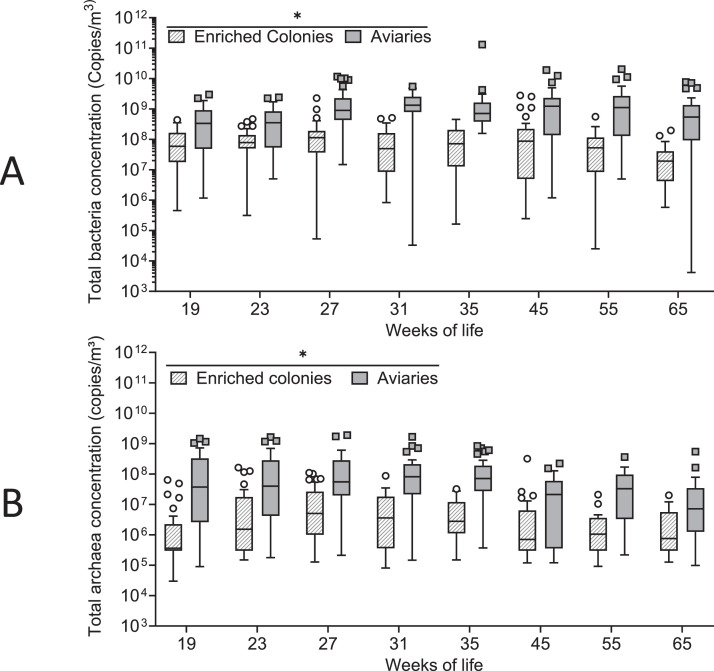
Concentrations of total bacteria (A) and archaea (B) in enriched colonies and aviaries during a full laying cycle, depending on the weeks of life (Median, Tukey, GLMM * = *p* < 0.05).

**Figure 2:**
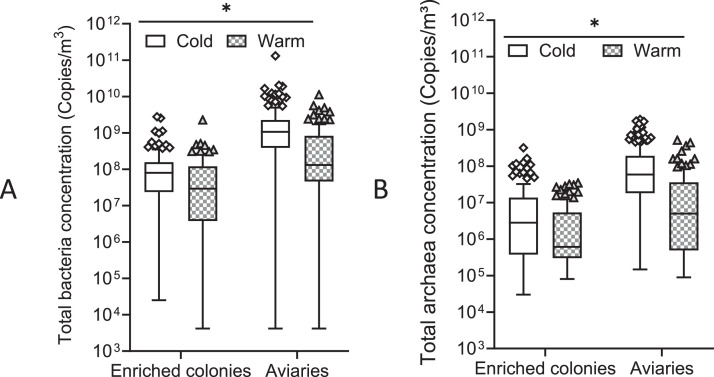
Concentrations of total bacteria (A) and archaea (B) during the cold and the warm seasons in enriched colonies and aviaries during a full laying cycle. (Median, Tukey, GLMM * = *p* < 0.05). Cold season: October to April, inclusively; Warm seasons: May to September, inclusively.

Concentrations of total bacteria in enriched colonies (n = 243 samples) ranged from 2.50 × 10^4^ to 2.77 × 10^9^ copies/m³, with the median value being 6.11 × 10^7^ copies/m³ (mean ± SD: 1.40 ± 3.11 × 10^8^ copies/m³), and in aviaries (269 samples), these ranged from 4.17 × 10^3^ to 1.31 × 10^11^, with the median value being 7.79 × 10^8^ copies/m³ (mean ± SD: 2.01 ± 8.30 × 10^9^ copies/m³) (Figure 1). Total bacterial concentrations in aviaries were significantly higher than in enriched cages (p < 0.001) and that in both setups were greater during the cold season (October through April) than during the warm season (May through September) (*p* = 0.020) (Figure 2). However, variables like flock age (*p* = 0.320), and months (*p* = 0.626) did not have a significant effect on total bacterial concentrations in either of the housing types (GLMMs, α = 0.05).

### Total Airborne Archaeal Concentrations

Concentrations of total archaea (Figure 1) were between 3.00 × 10^4^ and 3.19 × 10^8^ copies/m³ with a median of 1.73 × 10^7^ copies/m³ (mean ± SD: 1.10 ± 2.95 × 10^7^ copies/m³) in enriched colonies (235 samples). Whereas total archaeal concentrations in aviaries (269 samples) were between 9.00 × 10^4^ and 1.90 × 10^9^ copies/m³, with a median of 3.73 × 10^7^ copies/m³ (mean ± SD: 1.48 ± 2.98 × 10^8^ copies/m³). Total archaeal concentrations were significantly higher in aviaries compared to enriched cages (*p* = 0.007), as well as during the cold compared to the warm season (*p* = 0.009) in both facility types (Figure 2). Neither variables like flock age (*p* = 0.733) nor months (*p* = 0.060) had a significant effect on levels of total archaea (GLMMs, α = 0.05).

### Coverage of Bacterial and Alpha Diversity

Following quality control, removal of non-bacterial reads and of sequences present in blanks, an average of 18,702 reads were found in each sample, with a minimum of 2,041 reads and a maximum of 45,671 reads. All samples were rarefied to 2,041 sequences for each, for subsequent analyses. However, 2 samples, for which more than 40% of sequences were removed early during length filtration, and even from the data set (enriched colonies [5] – warm season, enriched colonies [10] – cold season).

**Figure 3:**
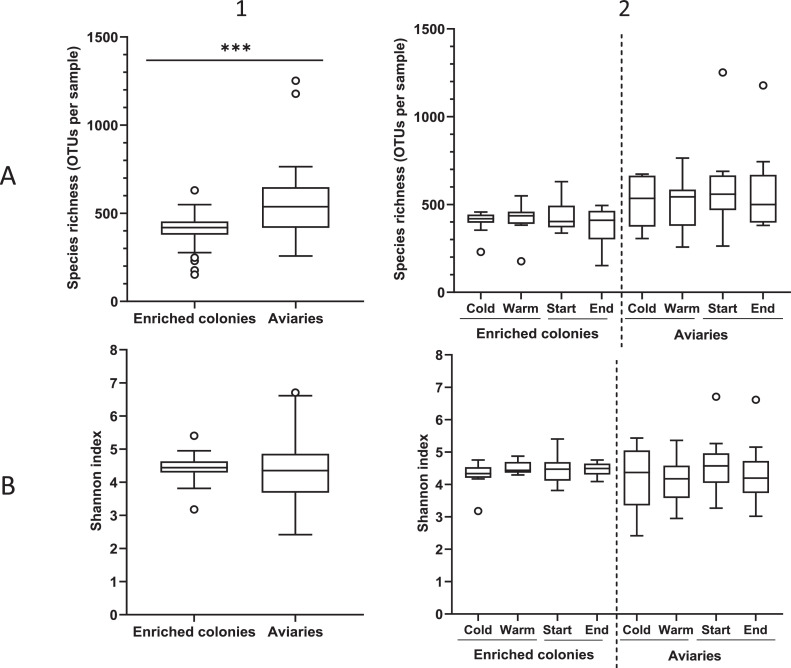
Species richness (A) and Shannon index (B) in enriched colonies and aviaries (1), during the cold/warm season and start/end of the laying cycle (2) (Median, Tukey, GLMM * = *p* < 0.05). Cold season : October to April, inclusively; Warm seasons: May to September, inclusively.

Figure 3 shows the specific richness of the samples (number of OTUs per sample) for both housing types, depending on the time in the production cycle and the season. A significant difference (Mann-Whitney, *p* < 0.001) was observed in species richness for enriched colonies (min: 152, max: 630, median: 419, mean: 407.3, SD: 87.63, n = 45) compared to that in aviaries (min: 258, max: 1252, median: 537, mean: 543.7, SD: 192.7). It was not statistically significant between the start or the end of the laying cycle or between the cold and the warm seasons for both setups (Wilcoxon and Student, *p* > 0.05). Shannon index values were not significantly different among all tested groups (Wilcoxon and Student, *p* > 0.05, Figure 3).

### Community Composition and Beta Diversity

**Figure 4:**
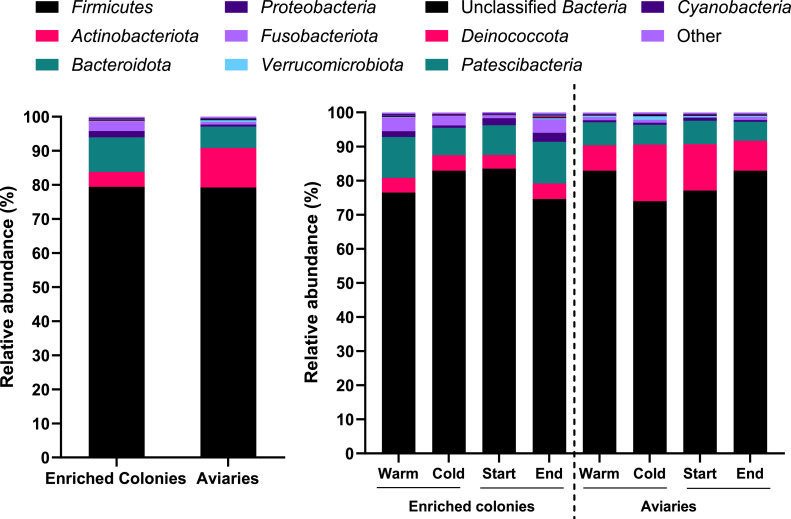
Relative abundance of the 10 most abundant bacterial phyla in enriched colonies and aviaries, depending on the sampling time and the season.

**Figure 5:**
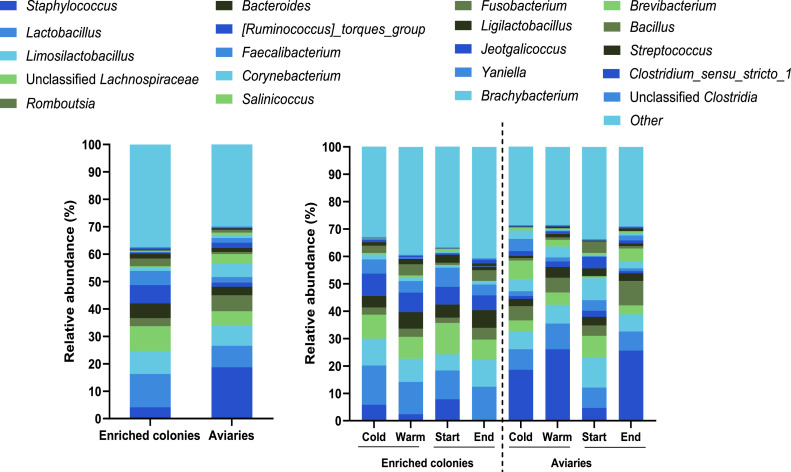
Relative abundance of the 20 most abundant bacterial genera in enriched colonies and aviaries, depending on the time in the production cycle and the season.

Figure 4 displays the 10 most abundant bacterial phyla and Figure 5 the 20 most abundant bacterial genera in each group.

*Firmicutes* were the dominant phylum in all groups, with a mean relative abundance ranging from 74.0% to 83.4%. *Actinobacteria* (3.94–16.6%) and *Bacteroidota* (5.56–12.2%) were the second most abundant. The other 7 were *Proteobacteria* (0.52–2.66 %), *Fusobacteriota* (0.17–4.02 %), *Verrucomicrobiota* (0.08–1.04 %), *Deinococcota* (0.02–0.28 %), *Patescibacteria* (0.02–0.320 %) and *Cyanobacteria* (0.22–0.47 %). Unclassified bacteria and other taxa respectively represented 0.08 to 0.32%, and 0.31 to 0.53%.

**Figure 6:**
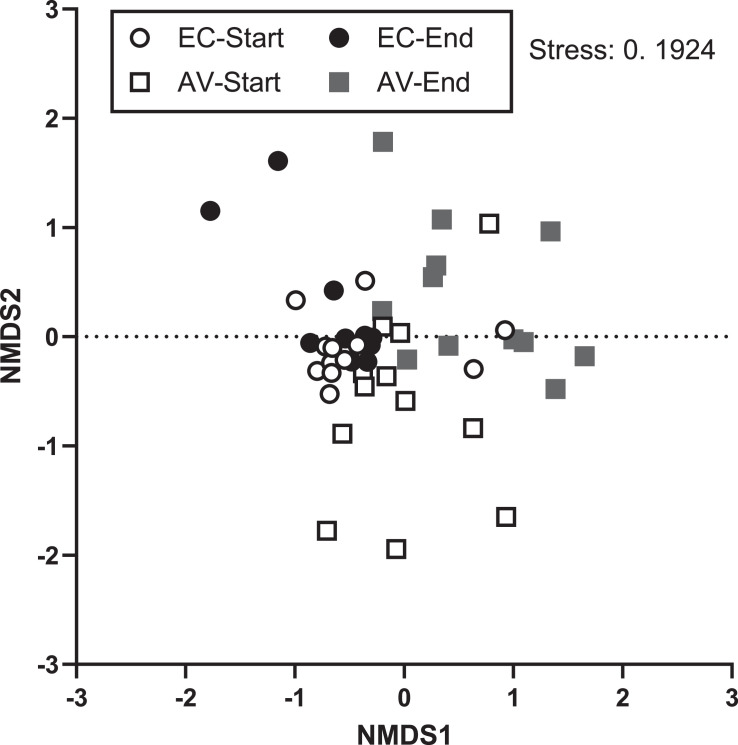
Non-metric multidimensional scaling (**NMDS**) of samples from enriched colonies (**EC**) and aviaries (**AV**), between the start and end of the laying cycle for each facility type (Bray-Curtis, Stress 0.1924).

Most abundant genera were *Staphylococcus* (0.18–26.1 %)*, Lactobacillus* (6.98–14.3 %) and *Limosilactobacillus* (5.84–11.0 %). The others were unclassified *Lachnospiraceae* (3.29–11.5 %)*, Romboutsia* (1.91–8.89 %)*, Bacteroides* (2.64–6.5 %)*,* [*Ruminococcus*] torques group (0.78–8.1 %), *Faecalibacterium* (0.96–6.97 %)*, Corynebacterium* (0.66–7.99%)*, Salinicoccus* (0–6.71 %)*, Fusobacterium* (0.17–4.02 %)*, Ligilatobacillus* (0.88–2.92 %)*, Jeotgalicoccus* (0.1–4.18 %)*, Yaniella* (0.02–4.39 %)*, Brachybacterium* (0–3.07 %)*, Brevibacterium* (0.05–1.24 %)*, Bacillus* (0–4.19 %)*, Streptococcus* (0.05–1.02 %)*, Clostridium sensus stricto 1* (0.09–1.42 %) and unclassified *Clostridia* (0.27–0.51 %)*.* Other genera represented approximately 28.5 to 40.7%. Bray-Curtis dissimilarity index computed from sample data indicates that bacterial community composition was significantly different between enriched colonies (**EC**) and aviaries (**AV**) (NPMANOVA, *p* = 0.001, Figure 6) and between the start and the end of the laying cycle for the 2 housing types (NPMANOVA, [EC] *p* = 0.003, [AV] *p* = 0.002, Figure 6). No significant differences were found among samples from cold and warm seasons, for both enriched colony (*p* = 0.989) and aviaries (*p* = 0.619).

Main OTUs contributing to the differences between enriched colonies and aviaries were unclassified *Lactobacillus*, [*Ruminococcus*] torques group, *Lactobacillus pontis*, uncultured *Faecalibacterium*, unclassified *Fusobacterium*, and unclassified *Romboutsia*, which were more abundant in enriched colonies while the taxa *Staphylococcus equorum, Staphylococcus lentus, Corynebacterium stationis*, unclassified *Jeotgalicoccus*, uncultured *Salinicoccus* and uncultured *Yaniella* being more abundant in aviaries (Table S1 in Supplementary material).

By contrast, the OTUs being more abundant at the start of the laying cycle in enriched colonies were [*Ruminococcus*] torques group, uncultured *Faecalibacterium, Staphylococcus equorum, Staphylococcus lentus,* unclassified *Blautia* and *Bacteroides caecigallinarum* while those at the end were identified as unclassified *Lactobacillus, Lactobacillus pontis*, unclassified Romboutsia, unclassified *Fusobacterium, Bacteroides barnesiae* and *Lactobacillus mucosae* (Table S2 in Supplementary material). Taxa that were the most abundant at the start of the laying cycle in aviaries were *Lactobacillus pontis, Corynebacterium stationis*, unclassified *Lactobacillus*, unclassified *Jeotgalicoccus,* uncultured *Faecalibacterium, Bacillus thermoamylovorans,* and *Lactobacillus aviaries*. Finally, the taxa unclassified *Romboutsia, Staphylococcus lentus, Staphylococcus equorum*, unclassified *Salinicoccus* and unclassified *Yaniella* were more abundant at the end of the production cycle in them (Table S3 in Supplementary material).

## DISCUSSION

The present study aimed to assess and compare concentrations of total airborne bacteria and archaea in twelve enriched colonies (enriched cages) and twelve aviaries for laying hens at different times during the production cycle (19, 23, 31, 35, 45, 55 and 65 wk of life). Moreover, airborne bacterial diversity was evaluated for both housing types, at the start and the end of the laying cycle, and in the cold and the warm seasons.

### Total Airborne Bacterial and Archaeal Concentrations

Concentrations of total airborne bacteria in enriched colonies (Figure 1) were similar to those previously found in others (furnished cages) through culture-independent methods for analysis (mean ± SD, 1.6 ± 0.55 × 10^7^ cells/m³) (Nimmermark et al., 2009), and in facilities with conventional cage systems (mean, 1.1 × 10^7^ *Escherichia coli* equivalents/m³) (Just et al., 2011). However, total airborne bacterial concentrations in aviaries (Figure 1) were slightly higher than those that were in cage-free housing systems for laying hens using multi-tier perches (mean ± SD, 2.8 ± 0.6 × 10^7^ cells/m³) (Nimmermark et al., 2009), but within the range reported for poultry operations (broilers, layers, turkeys) (from 2.7 × 10^7^ to 4.2 × 10^10^ cells/m³, with a mean of 4.7 × 10^9^ cells/m³) (Radon, 2001). Some work has investigated airborne archaea in laying hen houses. Concentrations in the present study were slightly higher than the mean concentration previously measured in facilities with conventional houses (1.2 × 10^6^ *Methanosarcina mazei* equivalents/m³) (Just et al., 2013).

Increased concentrations of both total airborne contaminants were observed in aviaries as expected. Those environments reportedly show higher concentrations of other bioaerosols such as particulate matter (Nimmermark et al., 2009; Le Bouquin et al., 2013) and endotoxins (Arteaga et al., 2015) than poultry operations with cage housing. The presence of soil and/or feces on the floor, the freedom of movement for birds and bioaerosol generating activities (foraging, scratching, and dust bathing) all contribute for air dispersion of organic matter and production of bioaerosols in aviary houses. Flock age had no significant effect on total airborne bacterial and archaeal concentrations that were reported in both enriched colonies and aviaries.

Previous positive correlations were discovered between the levels of airborne bacteria and aging birds in hatcheries and broiler operations (Bródka et al., 2012; Lawniczek-Walczyk et al., 2013), while Gustafsson and Wachenfelt could not find any association between the concentration of particulate matter and the age of laying hens (Gustafsson and Wachenfelt, 2006). It may be due to the hens usually keeping the same relative weight during the production cycle (Gustafsson and Wachenfelt, 2006), unlike broiler chickens, which can grow quite rapidly to the slaughter age. Higher levels of particulate matter and of other bioaerosols had previously been reported in poultry operations during colder seasons (Takai et al., 1998; Basinas et al., 2015), which are attributed to lower ventilation rates to preserve heat in buildings. Similar observations were made herein for the concentrations of both total airborne contaminants in enriched colonies and aviaries. However, as the level of ventilation was not assessed as part of this study, its contribution in the measuring of airborne bacterial and archaeal concentrations can only be inferred.

### Airborne Bacterial Diversity

Diversity of airborne bacteria in enriched colonies and aviaries was expected to reflect the main possible sources of bioaerosols inside facilities (e.g., birds, feed, and litter). The bacterial diversity in enriched cages was anticipated to be slightly different compared to aviaries, as the litter substrate present in the latter is unique to these environments and may not only contribute to the enrichment of specific bacteria but might also be an important source of them due to animal movement and their interactions with it.

Airborne bacteria communities in aviaries harbored a higher species richness (number of OTUs per sample) compared to enriched colonies (Figure 2). However, their respective Shannon index did not differ significantly. These results imply richness and evenness of the taxa that are similar in both housing types, despite having a larger number of OTUs per sample in aviaries. No significant difference was found between the 2 alpha-diversity indices when comparing the start/end of the laying cycle, the cold/warm seasons suggesting that neither the season nor the age of birds had an impact on the species richness nor the richness and evenness of the taxa.

The most abundant phyla found in enriched cages and aviaries (Figure 4) were consistent with previous analyses of bacterial diversity in laying hen houses (Cui et al., 2023), as *Bacillota (Firmicutes), Bacteroidota (Bacteroidetes), Proteobacteria, Cyanobacteria, Fusobacteriota (Fusobacteria)* and *Actinobacteriota (Actinobacteria)* were some in bioaerosols from laying hens. The high relative abundance of the phylum *Bacillota* (*Firmicutes)* in the air of laying hen houses may be due because it is very abundant in bird feces (Dai et al., 2022). *Bacteroidota (Bacteroidetes*), *Proteobacteria* and *Fusobacteria* were also dominant phyla in the gut microbiota of laying hens (Dai et al., 2022; Wang et al., 2023b) and during the peak egg production (30–50 weeks of living) (Dai et al., 2022).

The most dominant genera (Figure 5) were associated with gut, fecal or cecal microbiome of laying hens, which are *Lactobacillus, Limosilactobacillus,* unclassified *Lachnospiraceae, Romboutsia, Bacteroides,* [*Ruminococcus*] torques group, *Faecalibacterium, Salinicoccus, Fusobacterium, Ligilactobacilus*, and *Jeotgalicoccus* (Holdeman and Moore, 1974; Vos et al., 2009; Whitman et al., 2012; Khan et al., 2020; Rychlik, 2020; Zheng et al., 2020; Cui et al., 2023). The genus *Corynebacterium* is linked to the chicken gut and skin microbiome (Whitman et al., 2012; Cui et al., 2023)*,* while *Staphylococcus* and *Streptococcus* are involved with the skin and mucosal flora of warm-blooded animals (Vos et al., 2009). *Brevibacterium, Bacillus* and the *Clostridia* compose both skin or gut microbiome of birds as well as soil microflora (Vos et al., 2009; Whitman et al., 2012; Khan et al., 2020). Among the 20 most abundant genera, 3 were solely associated with soil and deep poultry litter, namely *Yaniella, Clostridium sensus stricto 1*, and *Brachybacterium* (Whitman et al., 2012; Li et al., 2023)*.* Main contributors to airborne bacterial diversity seem then to be feces, skin and mucosal flora of birds, and soil. As expected, significant differences in bacterial diversity were found between bioaerosols from enriched colonies and that from aviaries. Bacteria associated mostly with gut and fecal microbiome were more abundant in enriched cages, while those linked to gut, fecal and skin microbiome were in aviary houses. The presence of skin-associated bacteria might be due to the greater freedom of movement in cage-free facilities (flying, preening, dust bathing) that may contribute more to their aerosolization compared to enriched colonies. Variations in bacterial diversity between the start and the end of the production cycle were observed in both types of setups (Table S2 in Supplementary material). Bacteria from the genus *Staphylococcus* and fecal bacteria were initially found in higher abundance when at the laying cycle started in enriched colony housing, and then fecal bacteria became more abundant at its end. In aviaries, skin-associated bacteria had a same abundance whether the production cycle started or ended. The shift observed in enriched colonies may result from the initial gut and skin bacterial flora of birds, being overtaken by the flora originating from manure, as dust is becoming the main bioaerosol source. In contrast, original wood shavings as litter in aviaries is gradually replaced by manure, feathers, feed being dropped and other particulates like regular scrapings from floors.

The presence with higher abundance of *Staphylococcus lentus* and *Staphylococcus equorum* in bioaerosols may be due to the cumulative shedding of the skin from hens. Though the feces seem to be a main source of airborne bacteria in poultry operations, the longitudinal changes in the airborne bacterial flora did not fully overlap with the evolution of gut microbiota reported in active laying hens (Dai et al., 2022), as *Bacillota (Firmicutes)* remained the dominant phylum and did not get surpassed by *Bacteroidota* (*Bacteroidetes).*

### Strengths and Limitations of Study

The sampling in twelve enriched colonies and twelve aviaries, all were commercial, at multiple points over the laying cycle provided a large data set, enough to have a detailed picture of bioaerosols from these types of laying hen facilities. However, since the season and months were not controlled in the selection of barns and sampling, further study controlling for this factor along with hen age and housing type may be needed to assess the contribution and the possible interaction between hen age and the monthly or the seasonal effect. Measurements of ventilation rates at the time of sampling would also be relevant to assess its direct contribution on bioaerosol concentrations.

Since visited hen facilities, except one, was from the province of Québec (Eastern Canada, humid continental climate), one enriched cage setup being in Ontario had similar flock management practices, the airborne bacterial diversity revealed in the present study may not entirely reflect the one observed overall in the laying hen industry. The use of the molecular approach to quantify both bacteria and archaea allowed to bypass the limitation of opting for culture-dependent methods, since sampling can be stressful for microorganisms, and most bacterial populations cannot be grown and archaea cultivation can be fastidious (Delort, 2017). But qPCR still does not provide information about whether the quantified microbes are alive, or can cause infections. Amplicon sequencing was used to analyze bacterial diversity. However, the initial amplification of 16S rRNA genes may have led to a bias toward the most abundant taxa, so those that are seen less frequently may have been overlooked.

## CONCLUSIONS

High concentrations of airborne total bacteria and total archaea were found in both enriched colony and aviary houses, but in higher concentrations in aviaries than in enriched colonies. Higher concentrations for the 2 kinds of microorganisms were observed during cold seasons, while there was no significant longitudinal change for bacteria or archaea in both types of facilities. However, the bacterial diversity was significantly different between the start and the end of the laying cycle, whether it was enriched colonies or aviaries, which might indicate shifts in the bacterial flora because of animal activities and fecal accumulation. Despite slight overlaps in bacterial composition, diversity in enriched cages showed significant differences compared to aviaries.

The outcomes of this project give further insight on the concentrations of bacteria and archaea suspended in the air, and on the airborne bacterial diversity associated with 2 types of setups with alternative housing systems for laying hens. A better understanding of the bioaerosols in laying hen houses is necessary to study their effects on animal and human health. More work could aim at targeting bacterial or viral pathogens, and which could be disseminated as bioaerosols.

## DISCLOSURES

The authors declare no conflicts of interest.

## Appendix Supplementary materials

Image, application 1

## Footnotes

Supplementary material associated with this article can be found in the online version at doi:10.1016/j.psj.2024.104119.

## Footnotes

1 1981-2010 Climate Normals & Averages from weather stations closest to the sampling sites were used to determine the cutoff to distinguish the cold and the warm season (Government of Canada, 2021a). Chosen stations were : Montreal/St-Hubert A (Government of Canada, 2021e), Montreal/Mirabel INT'L A (Government of Canada, 2021d), St-Hyacinthe 2 (Government of Canada, 2021f), St-Wenceslas (Government of Canada, 2021b) and Ste-Anne-du-Lac (Government of Canada, 2021c).
