# Supplementary material for: Longitudinal survey of total airborne bacterial and archaeal concentrations and bacterial diversity in enriched colony housing and aviaries for laying hens
Source: Poult Sci. 2024 Jul 30;103(11):104119. doi: 10.1016/j.psj.2024.104119 (PMC11471094; doi:10.1016/j.psj.2024.104119)
Supplement: Supplementary file 1 [file mmc1.docx]

**Supplementary information**

For

**LONGITUDINAL SURVEY OF TOTAL AIRBORNE BACTERIAL AND ARCHAEAL CONCENTRATIONS AND BACTERIAL DIVERSITY IN ENRICHED COLONY HOUSING AND AVIARIES FOR LAYING HENS**

M-W St-Germain^*^, Valérie Létourneau^#^, Perrine Cruaud^##^, Candice Lemaille^*^, Kim Robitaille^*^, Éloïse Denis^§^, Martine Boulianne^§^, Caroline Duchaine^*,#^

^*^ Department of Biochemistry, Microbiology and Bioinformatics, Université Laval, Québec, Canada

# Research centre of Quebec Heart and Lung Institute, Quebec, Canada

# # Independent Researcher, Lourenties, France

^§^ Faculty of Veterinary Medicine, Université de Montréal, Saint-Hyacinthe, Canada

*Keywords*: bioaerosols, bacteria, archaea, layer, aviary, enriched colony, alternative housing systems

**Corresponding author:** Prof. Caroline Duchaine, [caroline.duchaine@bcm.ulaval.ca](mailto:caroline.duchaine@bcm.ulaval.ca)
2725, Chemin Sainte-Foy, Québec, Québec, G1V 4G5, CANADA

**Highlights:**

- Aviaries showed higher ambient concentrations of total bacteria and total archaea compared to enriched colonies
- Ambient concentrations of total bacteria and total archaea were higher during colder months (October to April), in aviaries and in enriched colonies
- Age of flocks did not have an effect on ambient concentrations of total bacteria nor total archaea, in aviaries and in enriched colonies

Housing type and flock age had an impact on the airborne bacterial diversity

| **Table S1. Mean abundance of the OTUs most responsible for significant differences between enriched colonies and aviaries**  (* : significant difference in abundance among groups, p < 0.05) | | |
| --- | --- | --- |
| **OTUs affiliation** | **% mean abundance**  **in enriched colonies** | **% mean abundance in aviaries** |
| Uncl. *Lactobacillus* (Firmicutes) | 10.79% * | 5.03% |
| [*Ruminococcus*] torques group (Firmicutes) | 6.31% * | 1.19% |
| *Lactobacillus pontis* (Firmicutes) | 5.08% * | 3.42% |
| Uncultured *Faecalibacterium* (Firmicutes) | 4.12% * | 1.45% |
| Uncl. *Fusobacterium* | 2.70% * | 0.70% |
| Uncl. *Romboutsia* (Firmicutes) | 2.91% | 5.45%* |
| *Staphylococcus equorum* (Firmicutes) | 2.29% | 11.82%* |
| *Staphylococcus lentus* (Firmicutes) | 0.93% | 2.67%* |
| *Corynebacterium stationis* (Actinobacteriota) | 0.67% | 3.57%* |
| Uncl. *Jeotgalicoccus* (Firmicutes) | 0.32% | 1.55%* |
| Uncultured *Salinicoccus* (Firmicutes) | 0.30% | 1.99%* |
| Uncultured *Yaniella* (Actinobacteriota) | 0.22% | 1.63%* |

| **Table S2. Mean abundance of the OTUs most responsible for significant differences between samples from the start of production cycles and samples from the end of production cycles in enriched colonies**  (* : significant difference in abundance among groups, p < 0.05) | | |
| --- | --- | --- |
| **OTUs affiliations** | **% mean abundance at the start of production cycles** | **% mean abundance at the end production cycles** |
| [*Ruminococcus*] torques group (Firmicutes) | 5.98%* | 5.04% |
| Uncultured *Faecalibacterium* (Firmicutes) | 5.43%* | 3.43% |
| *Staphylococcus equorum* (Firmicutes) | 3.59%* | 0.04% |
| *Staphylococcus lentus* (Firmicutes) | 2.05%* | 0 |
| Uncl. *Blautia* (Firmicutes) | 1.97%* | 1.13% |
| *Bacteroides caecigallinarum* (Bacteroidota) | 1.29%* | 0.50% |
| Uncl. *Lactobacillus* (Firmicutes) | 9.15% | 10.91%* |
| *Lactobacillus pontis* (Firmicutes) | 3.69% | 5.79%* |
| Uncl. *Romboutsia* (Firmicutes) | 1.88% | 4.23%* |
| Uncl. *Fusobacterium* | 0.80% | 3.84%* |
| *Bacteroides barnesiae* (Bacteroidota) | 0.38% | 1.48%* |
| *Lactobacillus mucosae* (Firmicutes) | 0.25% | 1.56%* |

| **Table S3. Mean abundance of the OTUs most responsible for significant differences between samples from the start of production cycles and samples from the end of production cycles in aviaries**  (* : significant difference in abundance among groups, p < 0.05) | | |
| --- | --- | --- |
| **OTUs affiliations** | **% mean abundance at the start of production cycles** | **% mean abundance at the end production cycles** |
| *Lactobacillus pontis* (Firmicutes) | 6.39%* | 2.18% |
| *Corynebacterium stationis* (Actinobacteriota) | 6.10%* | 1.48% |
| Uncl. *Lactobacillus* (Firmicutes) | 4.47%* | 4.09% |
| Uncl. *Jeotgalicoccus* (Firmicutes) | 3.42%* | 0.82% |
| Uncultured *Faecalibacterium* (Firmicutes) | 2.78%* | 0.66% |
| *Bacillus thermoamylovorans* (Firmicutes) | 2.72%* | 0.03% |
| *Lactobacillus aviarius* (Firmicutes) | 2.09%* | 0.07% |
| Uncl. *Romboutsia* (Firmicutes) | 3.64% | 8.33%* |
| *Staphylococcus lentus* (Firmicutes) | 1.22% | 3.20%* |
| *Staphylococcus equorum* (Firmicutes) | 1.19% | 16.16%* |
| Uncl. *Salinicoccus* (Firmicutes) | 0.33% | 2.09%* |
| Uncl. *Yaniella* (Actinobacteriota) | 0.30% | 1.75%* |
